# An experimental vignette study to assess stigmatized attitudes towards tobacco smokers in Kuwait

**DOI:** 10.18332/tpc/152254

**Published:** 2022-08-26

**Authors:** Shouq Al Dharman, Kawthar Safar, Fatima Al Enezi, Hessa Al Bahar, Alaa Ali, Zahra Al Qallaf, Athoub Al Shaya, Maryam Alenezi, Safiyah Al Otaibi, Maryam Al Nassar, Zainab Mohammad, Salman Alzayani

**Affiliations:** 1 Department of Preventive Medicine, Farwaniya Hospital, Ministry of Health, State of Kuwait; 2 Department of Otorhinolaryngology Head and Neck Surgery, Zain Hospital, Ministry of Health, State of Kuwait; 3 Farwaniya Hospital, Ministry of Health, State of Kuwait; 4 Ministry of Health, State of Kuwait; 5 Al Sabah Hospital, Ministry of Health, State of Kuwait; 6 Adan Hospital, Ministry of Health, State of Kuwait; 7 Department of Family and Community Medicine, College of Medicine and Medical Sciences, Arabian Gulf University, Kingdom of Bahrain

**Keywords:** tobacco, smoking, gender, attitude, stigma, Kuwait

## Abstract

**INTRODUCTION:**

Smoking is the most common method to consume tobacco. Although the prevalence of smoking is on the increase among females, it is still shown to be lower when compared with males, as there is a buildup of stigma towards tobacco smokers, with structural discrimination beginning to emerge. This study explored the effect of gender on stigmatizing attitude and behavior towards tobacco smokers.

**METHODS:**

An experimental vignette study design was used to explore the effect of gender on stigmatizing attitudes towards tobacco smokers of 151 students of both genders from Kuwait University. Students were divided into control and experimental groups and were provided with describing vignettes of male (control) and female (experimental) tobacco smokers along with the standard stigmatization questionnaire 1 (SSQ1). Data were analyzed using independent samples t-test, a p-value <0.05 was considered as statistically significant.

**RESULTS:**

Female smokers were more stigmatized than male smokers (p=0.007). In social self-interest, more students think that it is socially acceptable for men to smoke than it is for women (p<0.001). In evolutionary self-interest, there was a significant difference between the participants in accepting to marry or to have a relative who is a smoker (p<0.001), indicating disapproval for female smokers. In psychological self-interest, female tobacco smokers were not considered as good parents compared to male tobacco smokers (p=0.003).

**CONCLUSIONS:**

The findings of the study indicate the presence of stigmatizing attitudes towards female tobacco smokers in contrast to male tobacco smokers.

## INTRODUCTION

Smoking is the most common method to consume tobacco. In 2020, 22.3% of the global population used tobacco, 36.7% of all men and 7.8% of the women^1,2^. In high-income countries, the prevalence of male smokers is 35%, whereas the prevalence of female smokers is 22%. In Kuwait, the prevalence of male smokers is 39.2% and for females it is 3.3%^3,4^. Chronic exposure to tobacco is a risk factor for cardiovascular, lung and liver diseases that may lead to death^5^. Smoking is the second leading risk factor for early mortality and disability, globally^6^. Tobacco continues to adversely influence global health patterns, around 8 million people died from tobacco-related disease in 2017^2^. Tobacco smoking was found to be responsible for 16.3% of cancer cases in the Gulf Cooperation Council (GCC) countries^7^ (Bahrain, Kuwait, Oman, Qatar Saudi Arabia, and United Arab Emirates). In Kuwait, non-communicable diseases (NCDs) account for 72% of total deaths, and tobacco smoking is the third risk factor contributing to the four most common NCDs, namely cancer, diabetes, cardiovascular disease and chronic respiratory disease. The probability of dying between the ages of 30 and 70 years from the four main NCDs in Kuwait is 17%^8^.

The consequences of tobacco smoking have increased concern worldwide, forcing governments to put restriction laws in various settings, which had the outcome of preventing and decreasing the incidence of tobacco smoking. Although a great percentage of countries achieved significant rates of decrease in smoking prevalence, Kuwait remained one of four countries which had significant annual increases in smoking prevalence between 2005 and 2015, especially among women^9^. Although the prevalence of smoking is on the increase among females, it is still lower when compared with males, as there is a buildup of stigma towards tobacco smokers, with structural discrimination beginning to emerge in the context of tobacco smoking^10^. In some societies, it is believed that girls who smoke are seen as non-marriageable, which makes it unacceptable and uncommon for girls to smoke in public^11^.

Stigma is ‘an illuminating excursion into the situation of persons who are unable to confirm to the standards that the society calls normal’^12^. It causes someone to devalue or think less of the whole person. Stigma is classified into three types: the stigma of character traits, physical stigma, and the stigma of group identities like religion and race. Stigma processes can affect employment opportunities, housing, and access to medical care^13^.

Non-smokers perceive smokers as a minority with an anti-social and disapproved behavior, and consequently are regarded as ‘under-classed’ or are ‘blacklisted’. The idea of stigmatization is reinforced and demonstrated by the separation in public areas into smoker and non-smoker zones^14^. Friendships and marital relationships tend to be segregated with smoking behavior^15^. Non-smokers, the non-educated elderly, and those who abstained from alcohol are more prone to stigmatize smokers^16^. One of the undesirable effects of smoking stigma is the negative impact on smokers, by stereotyping them and reducing their self-esteem, leading to unsuccessful quitting attempts^17,18^.

In this study, we explored the effect of gender on stigmatizing attitudes and behaviors towards tobacco smokers among college students in Kuwait.

## METHODS

### Study design and instrument

**Figure 1:**
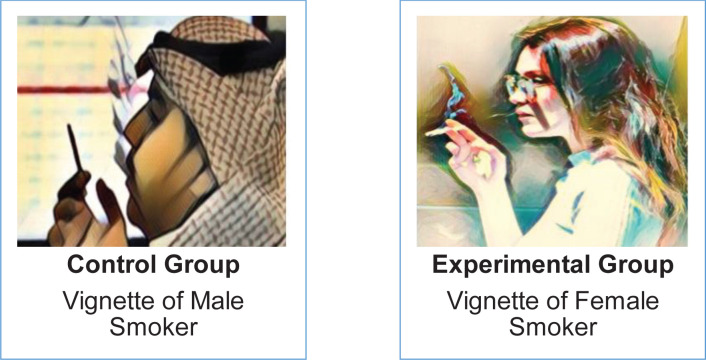
Smoker images presented to the study participants

This is an experimental vignette study that randomly assigns participants to two different groups; control and experimental, where different vignettes are used. The only expected difference between the control and the experimental groups is the outcome variable being studied. The study is designed to explore the effect of gender on stigmatizing attitudes and behaviors towards tobacco smokers. We provided each group with the same describing vignettes but with gender differences. The control group was provided with a male smoker vignette and image, while the experimental group was provided with a female smoker vignette and image (Figure 1) along with the standard stigmatization questionnaire 1 (SSQ1)^19^. The SSQ1 is used for assessing participants’ perception of stigmatization by others and participants’ predisposition to proceed to stigmatization. It contains questions that answer several factors which are social self-interest, evolutionary self-interest, and psychological self-interest. The words smoking, smoker and non-smoker were added to the questions to serve the research objective. We also provided all participants with a demographic data sheet that includes gender, age, and smoking status.

### Vignettes

Ahmad/Sarah is a 21-year-old engineering student in his/her 4th year. He/she is planning to work for an Oil Company after graduation. He/she smokes two packs of cigarettes per day. He/she has two sisters and one brother. He/she likes reading about sciences and listening to Arabic music.

### Study population

The study population was Kuwait University students attending the summer course. Data was collected from June to August 2017. Kuwait University is the primary public university in Kuwait, where the students who are enrolled are from all the six governorates of Kuwait. The colleges that were selected were: the College of Education, the College of Science, the College of Social Sciences, the College of Life Sciences, and the College of Engineering and Petroleum, because of the availability of the summer course during which our data collection was conducted. College of Sharia and Islamic studies was excluded to avoid biased results due to the fact that smoking is not favorable behavior from a religious perspective, while the College of Dentistry was excluded because the summer course is brief. The other colleges in Kuwait University do not provide summer courses. Students from other universities who were attending the summer courses at Kuwait University were excluded.

### Ethical considerations of the study

This study was approved by the Research and Ethics Committees of the College of Medicine and Medical Science at Arabian Gulf University (Approval number: E007-PI-6-17). Approval from Kuwait University was taken to distribute the questionnaire. Study participation was on a voluntary basis, and each participant was assured of complete confidentiality. All data were kept confidential.

### Sample size, randomization and data collection

A representative sample of students was divided into the control and experimental groups. The minimum number of subjects needed for medium-effect size was estimated as 64 subjects per group for the two experimental groups (n=128); we increased the sample size to 160 to ensure that the samples collected from each college had the same ratio of gender and each college subject was exposed to both vignettes. The 160 study subjects were further divided equally to 32 subjects collected from the five colleges. Our sample was divided into two groups; the first was the control where the participants were exposed to the control vignette (male tobacco smoker), while participants in the second group, the experimental group, were exposed to the experimental vignette (female tobacco smoker).

Consequently, we created two formats of our questionnaire; in order to ensure that 32 subjects taken from each college were exposed to the two formats, it was subdivided into four groups each having eight subjects. Each group included an equal ratio of female and male subjects which reflects the gender distribution in the population of interest. The female to male ratio was approximately 3:1. Three of the subjects did not return the questionnaire back to the researchers. Six different subjects had three or more missing values in their questionnaire. Thus, they were excluded from the study, yielding 151 subjects who were considered for analysis. The sampling technique that was used in this study is convenience sampling. Data collection was conducted by the research team, with the consent from each participant.

### Data analysis

Data entry was done using IBM SPSS Statistics. An independent sample t-test was used to determine whether there was statistical evidence that the associated population means were significantly different. The questionnaire consisted of 18 questions, each question had four possible responses: Definitely yes; Perhaps yes; Perhaps no; Definitely no. The answers to these questions were given a value, depending on the question. A score of 1 was given to the least and a score of 4 to the most perception of stigmatization. The higher the value the more the stigmatizing the attitude^19^. Using SPSS, we summed up the questions creating a new variable, the stigma level (SL) and an independent sample t-test was conducted. The independent variable was gender and the dependent variable was the SL. A p-value of <0.05 was considered statistically significant. We assessed the effect of stigma using this test to examine if there was a significant difference.

## RESULTS

### Demographic characteristics of the students

A total of 151 students were included in the study, of which 113 students were females and 38 students were males representing the 3:1 (female-to-male) ratio in Kuwait University during the summer course. The participants were equally distributed into the control (50.3%) and experiment (49.6%) groups. The mean age of the participants was 20.8 years; 57 were aged <20 years, 54 were 20–22 years and 18 were ≥23 years. Citizens of the GCC countries represented 95.3% of the study population. Students who had never smoked were 126, and 14 students smoked every day. Percentages of daily smokers, occasional smokers, ex-smokers, and never smokers were: 9.4%, 4.6%, 2.6%, and 83.4%, respectively (Supplementary file).

### Social self-interest

Social self-interest factor results (Table 1) show that there was a significant difference (p<0.001) between male and female smokers (Question 1), as more students thought that it is socially acceptable for men to smoke than it is for women. The participants also showed different attitudes (p=0.017) towards smokers sitting beside them (Question 4).

**Table 1:** Comparing social self-interest factor results between the control and experimental group, Kuwait University, 2017 (N=151)

*Questions*	*Answers*	*Control group*	*Experimental group*	*p*
		*n (%)*	*n (%)*	
1. Did you think he/she is socially accepted?	Definitely yes	40 (52.6)	9 (12.0)	0.000
	Perhaps yes	30 (39.5)	33 (44.0)	
	Perhaps no	3 (3.9)	12 (16.0)	
	Definitely no	3 (3.9)	21 (28.0)	
2. Would you avoid talking to him/her if possible?	Definitely yes	27 (36.0)	36 (48.0)	0.503
	Perhaps yes	19 (25.3)	12 (16.0)	
	Perhaps no	20 (26.7)	16 (21.3)	
	Definitely no	9 (12.0)	11 (14.7)	
3. Would you agree to sit next to him/her?	Definitely yes	22 (28.9)	30 (40.0)	0.323
	Perhaps yes	22 (28.9)	19 (25.3)	
	Perhaps no	17 (22.4)	11 (14.7)	
	Definitely no	15 (19.7)	15 (20)	
4. If he/she set beside you in class?	Start a conversation and be friendly	22 (28.9)	33 (44.0)	0.017
	Keep it quiet until the class starts	28 (36.8)	24 (32.0)	
	Ignore him/her	13 (17.1)	15 (20.0)	
	Change your seat	13 (17.1)	3 (4.0)	
5. Would you accept to eat food, which he/she has cooked?	Definitely yes	28 (36.8)	28 (37.3)	0.871
	Perhaps yes	31 (40.8)	29 (38.7)	
	Perhaps no	8 (10.5)	8 (10.7)	
	Definitely no	9 (11.8)	10 (13.3)	

### Evolutionary self-interest

Evolutionary self-interest factor results (Table 2) show a significant difference (p=0.000) between the students’ acceptance of a smoker marrying a relative (Question 4), indicating more rejection of the female smoker. It was more acceptable for a male teacher or care provider for children to be a smoker, Question 5 (p=0.001) and Question 6 (p<0.001) respectively, compared to their female counterparts. In addition, a trend was observed between the participants in Question 7 (p=0.098) and Question 9 (p=0.092). The statistical trend favors employment of male smokers over female smokers with the same qualifications in Question 7. Gender of smoker played a factor in Question 9 in the study population point of view prioritizing medical care.

**Table 2:** Comparing evolutionary self-interest factor results between the control and experimental group, Kuwait University, 2017 (N=151)

*Questions*	*Answers*	*Control group*	*Experimental group*	*p*
		*n (%)*	*n (%)*	
1. Can you be a friend of his/hers?	Definitely yes	17 (22.4)	20 (26.7)	0.187
	Perhaps yes	40 (52.6)	18 (24.0)	
	Perhaps no	0 (0)	15 (20.0)	
	Definitely no	19 (25.0)	22 (29.3)	
2. Would you accept to work with him/her?	Definitely yes	36 (47.4)	34 (45.3)	0.231
	Perhaps yes	31 (40.8)	26 (34.7)	
	Perhaps no	7 (9.2)	8 (10.7)	
	Definitely no	2 (2.6)	7 (9.3)	
3. Would you be frightened if he/she came to live next door to you?	Definitely yes	45 (59.2)	44 (58.7)	0.953
	Perhaps yes	16 (21.1)	14 (18.7)	
	Perhaps no	9 (11.8)	13 (17.3)	
	Definitely no	6 (7.9)	4 (5.3)	
4. Will you be ok to marry or accept if your relative marries him/her?	Definitely yes	21 (27.6)	6 (8.0)	0.000
	Perhaps yes	27 (35.5)	16 (21.3)	
	Perhaps no	13 (17.1)	12 (16.0)	
	Definitely no	15 (19.7)	41 (54.7)	
5. Would you accept if he/she became the teacher of your children?	Definitely yes	21 (27.6)	12 (16.0)	0.001
	Perhaps yes	29 (38.2)	20 (26.7)	
	Perhaps no	11 (14.5)	10 (13.3)	
	Definitely no	15 (19.7)	33 (44.0)	
6. Would you allow him/her to take care of your children or younger relatives?	Definitely yes	17 (22.4)	7 (9.3)	0.000
	Perhaps yes	29 (38.2)	21 (28.0)	
	Perhaps no	12 (15.8)	9 (12.0)	
	Definitely no	18 (23.7)	38 (50.7)	
7. If you were an employer, would a non-smoker have an advantage compared to him/her if they had the exact resume?	Advantage for the non-smoker	5 (6.6)	2 (2.7)	0.098
	Advantage for the smoker	48 (63.3)	42 (56.0)	
	No difference	23 (30.3)	31 (41.3)	
8. If you had to work with him/her in a project/assignment; how would you interact?	Friendly and maybe start a friendship	32 (43.2)	23 (30.7)	0.102
	Colleague and keep it professional	41 (55.4)	50 (66.7)	
	Ask the teacher/Dr/Professor to change the partner	1 (1.4)	2 (2.7)	
9. If he/she and another individual who is a non-smoker had the same disease; which one has more privilege to be treated first?	The non-smoker	6 (7.9)	9 (12.0)	0.092
	The smoker	57 (75.0)	57 (76.0)	
	Both	13 (17.1)	9 (12.0)	

### Psychological self-interest

The results of the psychological self-interest factor are presented in Table 3. There was a significance difference (p=0.003) between the students’ opinions regarding male and female smokers being parents. There were no other significant differences between the other questions assessing the psychological factor.

**Table 3:** Comparing psychological self-interest factor results between the control and experimental group, Kuwait University, 2017 (N=151)

*Questions*	*Answers*	*Control group*	*Experimental group*	*p*
		*n (%)*	*n (%)*	
1. Would you think he/she is a bad person?	Definitely yes	28 (36.8)	19 (25.3)	0.197
	Perhaps yes	26 (34.2)	28 (37.3)	
	Perhaps no	18 (23.7)	25 (33.3)	
	Definitely no	4 (5.3)	3 (4)	
2. Do you agree that he/she smokes to deal with his daily life?	Definitely yes	15 (19.7)	17 (22.7)	0.257
	Perhaps yes	18 (23.7)	21 (28.0)	
	Perhaps no	30 (39.5)	30 (40.0)	
	Definitely no	13 (17.1)	7 (9.3)	
3. Do you think he/she will be a good parent?	Definitely yes	21 (27.6)	6 (8.0)	0.003
	Perhaps yes	40 (52.6)	44 (61.3)	
	Perhaps no	11 (14.5)	11 (14.7)	
	Definitely no	4 (5.3)	12 (16.0)	
4. Do you think that he/she would benefit from counseling or therapy to quit smoking?	Definitely yes	5 (6.6)	-	0.137
	Perhaps yes	3 (3.9)	4 (5.3)	
	Perhaps no	31 (40.8)	30 (40.0)	
	Definitely no	37 (48.7)	41 (54.7)	

### Total stigma level (SL)

The total stigma level (SL), derived from the sum of the questions, between the control and experimental groups was significant (p=0.007).

## DISCUSSION

This study is one of the first studies addressing tobacco smoking stigma in the Arabian Gulf Region, and it has several strong points such as using vignettes to study stigma, its experimental design which is not well explored in the region and the world, population of the study are millennials, and the questionnaire used addressed stigma from several aspects. This study helped in highlighting the effect of gender on smoking stigma and understanding the power of stigma toward smokers. The study emphasized stigmatizing attitudes and behaviors towards tobacco smokers in the community, as this would increase the awareness among smokers and it might be a reason for them to quit.

The results showed a significant total stigma level between the control and experimental groups. Addressing the social aspects of the study, there was a significant difference between the students towards male and female tobacco smokers, as more students think that it is socially acceptable for men to smoke than it is for women. The findings addressing the social self-interest factor towards tobacco smokers suggest that female smokers are socially not accepted compared to males. The disapproval of female smokers is imprinted in the societies of GCC countries and that socially accepting smokers is determined by gender. The results of the evolutionary self-interest factor showed a significant difference between the participants in accepting to marry or have a relative who is a smoker, indicating disapproval for female smokers. In addition, gender seems to be a significant factor when the teacher is a smoker and when a smoker takes care of children and young relatives. Although the study sample reflects that the community perceives female smokers to have less healthcare rights, we believe that actual medical practice in Kuwait does not parallel this finding. Moreover, the psychological self-interest factor results showed a significant difference between the students’ opinions regarding the qualification of tobacco smokers for being parents, where results showed that female tobacco smokers could not be a good parent compared to male tobacco smokers. This could be explained with female smokers crossing the norm line in the society and the concept of smoking being a gate for drug addiction. In contrast, in other aspects of the study questionnaire, gender had no observed effect on subjects’ bias against smokers, like sitting next to them, working with them, or eating food they have cooked. This would also be explained by the psychological self-interest factor results, which demonstrated that there was no pre-judgment of a smoker’s character. Moreover, unlike marriage that showed significant disapproval of female smokers, those mentioned encounters tend to be brief and short-lasting compared to marriage associated with a long, deep relationship. Similar results were found in studies from other parts of the world^20-22^.

Kuwait has invested in smoking cessation due to its determinantal effect on public health. It is prohibited to sell or offer cigarettes, types of tobacco and its derivatives to anyone aged <21 years. Smoking is prohibited in public places to be specified by a decision of the Minister of Public Health, and the decision may specify the smoking places designated there. It is prohibited for workers in food stores to smoke while preparing food or drinks that are served to customers, and it is also prohibited to smoke while driving a car or any means within public or private transportation. Publicity and advertisement of cigarettes and types of tobacco and its derivatives are prohibited in the country. Every violation of the provisions of this law shall be punished by a fine not exceeding 50 Kuwaiti dinars (about 163 US$), and the penalty shall be doubled in case of recurrence. In 2021, the Kuwait Ministry of Health revealed their plan to open 50 clinics for quitting smoking in primary healthcare centers and hospitals in the upcoming five years, 10 clinics annually with strong infrastructure. Health Ministry launched several extensive media campaigns on stopping the consumption of all types of tobacco. Mortality and morbidity are increased by three times compared to non-smokers, increasing from low to high, but females in both are more affected^23^. It has been established that the smoking stigma has undesirable effects which can negatively impact cessation attempts^17,18^. Smokers’ stigma does not exist in isolation and is influenced by one’s social identity such as gender, race, and ethnicity. For a smoker whose stress relief is cigarette smoking, structural oppression can cause further stress leading to social isolation and marginalization^24^. Smoking’s perceived risks and benefits are linked with intentions to quit and vary between genders^25^. Kuwait’s and the region’s further policies should target different demographic profiles with various tools to achieve a long-term effect of smokers’ absence. In the UAE, another neighboring country with a similar demographic status, female adolescent smoking was the highest among those of Arab/Middle-Eastern descent^26^.

## CONCLUSIONS

The findings of the study indicate the presence of stigmatizing attitudes towards female tobacco smokers in contrast to male tobacco smokers. New policies of smoking control should take into consideration gender variation and ethnicity. We suggest applying more research in a wider and diverse population to reflect the stigmatizing attitude towards female tobacco smokers of different ethnical backgrounds. Different types of studies with stronger design such as randomized control trails should be conducted in further studies.

## Data Availability

The data supporting this research are available from the authors on reasonable request.
