# Supplementary material for: An experimental vignette study to assess stigmatized attitudes towards tobacco smokers in Kuwait
Source: Tob Prev Cessat. 2022 Aug 26;8:32. doi: 10.18332/tpc/152254 (PMC9414082; doi:10.18332/tpc/152254)
Supplement: Supplementary file 1 [file TPC-8-32-s1.pdf]

Supplementary Table: Demographic Characteristics of the Students

Demographic Data		Control Group	Experimental Group
		N (%)	N (%)
Gender	Male	19 (25)	19 (25.3)
	Female	57 (75)	56 (74.7)
	Total	76 (100)	75 (100)
Age Group	<20	31 (50.8)	26 (38.2)
	20-22	23 (37.7)	31 (45.6)
	≥23	7 (11.5)	11 (16.2)
Nationality	GCC	72 (96.0)	72 (97.3)
	Non-GCC	3 (4)	2 (2.7)
Smoking Status	I have never smoked	62 (81.6)	64 (85.3)
	I have quit smoking	2 (2.6)	2 (2.7)
	I smoke occasionally	3 (3.9)	4 (5.3)
	I smoke every day	9 (11.8)	5 (6.7)

© 2022 Al Dharman S. et al.
